# Distribution patterns of 21-gene recurrence score in 980 Chinese estrogen receptor-positive, HER2-negative early breast cancer patients

**DOI:** 10.18632/oncotarget.16313

**Published:** 2017-03-17

**Authors:** Jiayi Wu, Yan Fang, Lin Lin, Xiaochun Fei, Weiqi Gao, Siji Zhu, Yu Zong, Xiaosong Chen, Ou Huang, Jian-Rong He, Li Zhu, Weiguo Chen, Yafen Li, Kunwei Shen

**Affiliations:** ^1^ Comprehensive Breast Health Center, Ruijin Hospital, Shanghai Jiaotong University School of Medicine, Shanghai 200025, China; ^2^ Department of Clinical Laboratory, Ruijin Hospital, Shanghai Jiaotong University School of Medicine, Shanghai 200025, China; ^3^ Department of Pathology, Ruijin Hospital, Shanghai Jiaotong University School of Medicine, Shanghai 200025, China

**Keywords:** breast carcinoma, 21-gene, risk score, clinico-pathologic factors

## Abstract

**Aim:**

The current study aimed to explore the distribution patterns of 21-gene recurrence score (RS) assay in Chinese early breast cancer patients.

**Methods:**

Nine hundred and eighty consecutive estrogen receptor(ER)-positive, human epidermal growth factor receptor 2 (HER2)-negative early breast cancer patients treated at Ruijin Hospital, Shanghai Jiaotong University, School of Medicine from 2009 to 2016 were retrospectively recruited. Reverse transcriptase-polymerase chain reaction (RT-PCR) assay of 21 genes were conducted in paraffin-embedded tumor tissue to calculate the RS. Co-relations of RS and clinico-pathologic factors were evaluated. Concordances of RT-PCR and immunohistochemistry (IHC) tests were measured. Logistic regression were applied to determine independent variables associated with RS.

**Results:**

The median RS of 980 patients was 23(0~90), and the proportions of patients categorized as having a low, intermediate, or high risk were 26.1%, 49.3% and 24.6%. The distribution of RS varied significantly according to different tumor grade, T stage, progesterone receptor(PR) status, Ki67 index and molecular subtypes (p<0.05). Grade, PR status and Ki67 index were identified as independent variables associated with RS. The concordance rates between RT-PCR and IHC test were 98.8% and 88.3% for ER and PR status, and there were weak to moderate correlation between IHC and RT-PCR tests for ER, PR expression and Ki67 index.

**Conclusions:**

RS correlated significantly with grade, T stage, PR status, Ki67 index and molecular subtypes in Chinese early breast cancer patients. Grade, PR status and Ki67 index could independently predict RS. ER, PR status and Ki67 index between RT-PCR and IHC test had remarkable concordance.

## INTRODUCTION

Breast cancer is now recognized as a group of disease with significant heterogeneity, and traditional clinico-pathologic factors were no longer enough to meet the needs of prognosis prediction and treatment decision. In 2000, Perou et al characterized variation in gene expression patterns in a set of 65 breast tumor specimens using complementary DNA microarrays, and classified breast cancer into five intrinsic subtypes [1]. In 2012, The Cancer Genome Atlas Network (TCGA) produced a comprehensive catalogue of likely genomic drivers of the most common breast cancer subtypes [2]. Up to now, the management of breast cancer has entered into the era of molecular subtype with gene expression profiling.

Over the past decade, several multigene assays, based on findings of previous gene expression profiling, were developed and applied into routine practice of estrogen receptor(ER)-positive breast cancer. Among them, 21-gene recurrence score (RS, Oncotype DX) assay had gained most sufficient analytical as well as clinical validity [3–12]. 21-gene RS is a quantitative reverse transcriptase polymerase chain reaction (RT-PCR)–based test measuring 21 genes in formalin-fixed paraffin-embedded breast tumors. Retrospective studies from the NSABP B-14 showed that the RS predicted the likelihood of distant recurrence or breast cancer death in patients treated with endocrine therapy alone [3]. The ability of RS to predict benefit from adjuvant chemotherapy in node-negative or 1~3 node-positive patients was confirmed by retrospective analysis of the NSABP B-20 and SWOG-8814 trials [4, 5]. Furthermore, the latest prospective validation of RS in TAILORx (Trial Assigning IndividuaLized Options for Treatment) and West German Study Group PlanB trial revealed that among patients with hormone receptor (HR)-positive, human epidermal growth factor receptor2 (HER2)-negative, node-negative breast cancer and not treated with adjuvant chemotherapy, those with tumors that had a low-risk RS had very low rates of recurrence [11, 12].

Based on these findings, RS has changed patient treatment recommendation in 20% to 70% of cases and has resulted in a 13% to 34% reduction in adjuvant chemotherapy [13]. RS is now the only genomic test for breast cancer recommended by National Comprehensive Cancer Network (NCCN) which has the ability to predict response to adjuvant treatment [14]. However, due to the paucity of data in China, clinical application of RS in Chinese breast cancer patients remained unclear. The current study aimed to evaluate the distribution patterns of 21-gene RS in Chinese ER-positive, HER2-negative early breast cancer patients.

## RESULTS

### Distribution of RS

The baseline clinico-pathological features of the overall population are shown in Table 1. The median age at initial diagnosis was 56 years old (range: 27-93). Mastectomy was performed in 534(59.8%) patients. Seven hundred and ninety-five patients (88.7%) were diagnosed to have invasive ductal carcinoma (Table 2), and grade 1, 2, 3 tumors were documented in 17.0%, 57.9% and 25.1% of the patients. T1 and N0 tumors comprised 68.7% and 86.7% of all patients. Twenty-four patients (2.4%) had micrometastatic disease in their lymph nodes, and this part of patients was integrated into N0 subgroup for statistical analysis in the following part of the study.

**Table 1:** Characteristics of 980 eligible patients

Characteristics	N	%	Valid %
Age			
≤55	442	45.1	49.3
>55	454	46.3	50.7
Unknown	84	8.6	
Surgery			
Mastectomy	534	54.5	59.8
BCS	359	36.6	40.2
Unknown	87	8.9	
Pathology			
IDC	795	81.1	88.7
Others	101	10.3	11.3
Unknown	84	8.6	
Grade			
I	135	13.8	17.0
II	460	46.9	57.9
III	199	20.3	25.1
Unknown	186	19.0	
T stage			
T1	608	62.0	68.7
T2-3	277	28.3	31.3
Unknown	95	9.7	
N stage			
N0	850	86.7	86.7
N1mi	24	2.4	2.4
N1	106	10.8	10.8
ER			
Positive	980	100	100
Negative	0	0	0
PR			
Positive	750	76.5	83.8
Negative	145	14.8	16.2
Unknown	85	8.7	
Ki67			
<14	430	43.9	49.7
≥14	436	44.5	50.3
Unknown	114	11.6	
HER2			
Positive	0	0	0
Negative	980	100	100
Subtype			
Luminal A-like	288	29.4	33.3
Luminal B-like	578	59.0	66.7
Unknown	114	11.6	
RS category			
Low risk	256	26.1	26.1
Intermediate risk	483	49.3	49.3
High risk	241	24.6	24.6

Abbreviations: BCS, breast conserving surgery; IDC, invasive ductal carcinoma; ER, estrogen receptor; PR, progesterone receptor; HER2, human epidermal growth receptor 2; RS, recurrence score.

**Table 2:** Pathology of 980 eligible patients

Pathologic type	N	%	Valid %
Invasive ductal carcinoma	795	81.1	88.7
Mucinous carcinoma	50	5.1	5.6
Invasive lobular carcinoma	33	3.4	3.7
Invasive papillary carcinoma	7	0.7	0.8
Invasive micropapillary carcinoma	5	0.5	0.6
Neuroendocrine carcinoma	3	0.3	0.3
Tubular carcinoma	2	0.2	0.2
Medullary carcinoma	1	0.1	0.1
Unknown	84	8.6	
Total	980	100	100

**Figure 1:**
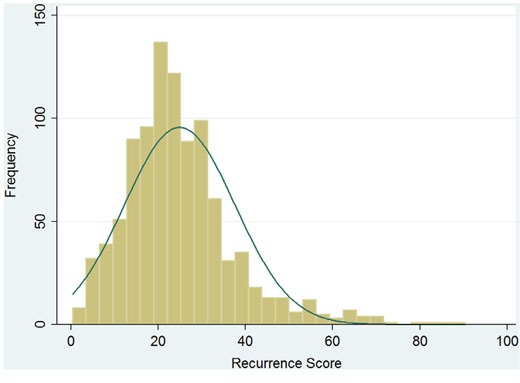
Recurrence score distribution of 980 eligible patients No captions.

Of the 980 patient, median RS was 23 (range: 0~90), and RS followed a skewed distribution (Kolmogorov and Shapiro test, p<0.001, Figure 1). There were 256 patients (26.1%) in the low-risk group (RS<18), 483 patients (49.3%) in the intermediate-risk group (RS 18 to 30), and 241 patients (24.6%) in the high-risk group (RS≥31).

**Figure 2:**
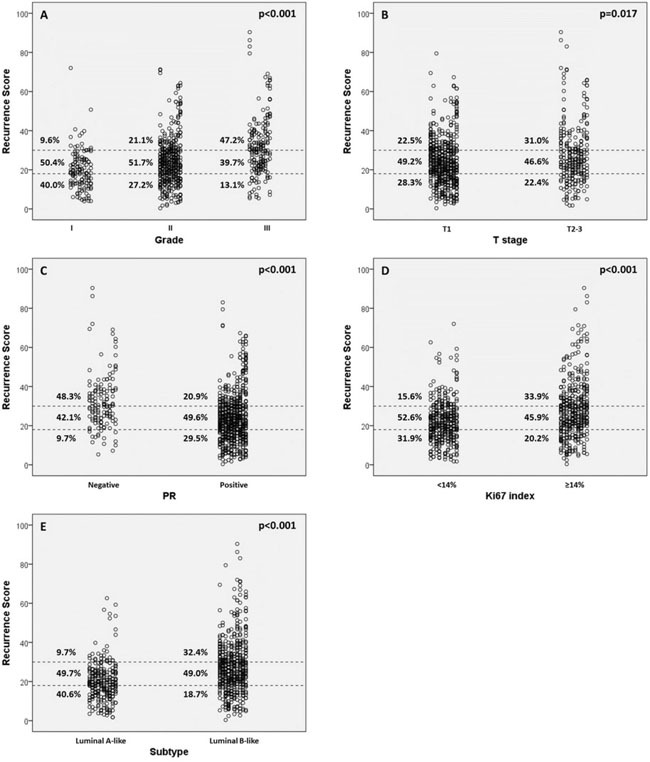
Recurrence score (RS) categories *vs*. clinic-pathologic factors **(A)** RS *vs* grade; **(B)** RS *vs* clinical t stage; **(C)** RS *vs* progesterone receptor (PR); **(D)** RS *vs* Ki-67 index; **(E)** RS *vs* molecular subtype.

The distribution of RS varied significantly according to different tumor grade, T stage, PR status, Ki67 index and molecular subtypes (all p<0.05, Table 3). High grade, large tumor size, negative PR, high Ki67 index and luminal B subtype were more likely to have high-risk RSs (Figure 2). In grade 1 tumors, the proportion of low, intermediate, or high-risk patients were 40.0%, 50.4%, and 9.6%, whereas it was 13.1%, 39.7% and 47.2% in patients with grade 3 tumors (p<0.001). Likewise, T1, PR-positive and low-Ki67 tumors were more likely to be categorized as low or intermediate risk and less likely to be categorized as high risk compared with T2-3, PR-negative and high-Ki67 tumors (all p<0.05).

**Table 3:** Distribution of RS according to clinico-pathologic factors

Characteristics	Total N	RS category, N (%)			P value
		Low risk	Intermediate risk	High risk	
Age	896				0.325
≤55		106(24.0)	221(50.0)	115(26.0)	
>55		129(28.4)	213(46.9)	112(24.7)	
Surgery	893				0.167
Mastectomy		152(28.5)	254(47.6)	128(24.0)	
BCS		83(23.1)	177(49.3)	99(27.6)	
Pathology	896				0.321
IDC		204(25.7)	384(48.3)	207(26.0)	
Others		31(30.7)	50(49.5)	20(19.8)	
Grade	794				<0.001
I		54(40.0)	68(50.4)	13(9.6)	
II		125(27.2)	238(51.7)	97(21.1)	
III		26(13.1)	79(39.7)	94(47.2)	
T stage	885				0.017
T1		172(28.3)	299(49.2)	137(22.5)	
T2-3		62(22.4)	129(46.6)	86(31.0)	
N stage	980				0.686
N0		232(26.5)	429(49.1)	213(24.4)	
N1		24(22.6)	54(50.9)	28(26.4)	
PR	895				<0.001
Positive		221(29.5)	372(49.6)	157(20.9)	
Negative		14(9.7)	61(42.1)	70(48.3)	
Ki67	866				<0.001
<14		137(31.9)	226(52.6)	67(15.6)	
≥14		88(20.2)	200(45.9)	148(33.9)	
Subtype	866				<0.001
Luminal A-like		117(40.6)	143(49.7)	28(9.7)	
Luminal B-like		108(18.7)	283(49.0)	187(32.4)	

Abbreviations: BCS, breast conserving surgery; IDC, invasive ductal carcinoma; PR, progesterone receptor; RS, recurrence score.

Among node-negative patients, all these factors remained significantly associated with RS (all p<0.05), whereas in 1~3 node-positive patients, T stage no longer had a significant correlation with RS.

### Multivariate analysis

Logistic regression analysis demonstrated that grade, PR, and Ki67 were independent variables associated with RS (Table 4). Grade 2 and 3 were associated with significantly higher odds of intermediate(Grade 2 vs Grade 1, OR: 1.576, 95%CI: 1.006~2.470, p=0.047; Grade 3 vs Grade 1, OR: 2.607, 95%CI: 1.363~4.988, p=0.004) and high-risk(Grade 2 vs Grade 1, OR: 2.709, 95%CI: 1.309~5.606, p=0.007; Grade 3 vs Grade 1, OR: 10.035, 95%CI: 4.222~23.854, p<0.001) RSs. Patients with PR-negative tumors were more likely to have intermediate (OR: 4.397, 95%CI: 1.952~9.901, p<0.001) and high-risk (OR: 12.039, 95%CI: 5.207~27.836, p<0.001) RSs compared with those with PR-positive tumors. In addition, proportion of high-risk RS was also significantly higher among patients with high Ki67 index (OR: 1.719, 95%CI: 1.051~2.812, p=0.031). Tumor size had no significant impact on RS categories.

**Table 4:** Multivariate analysis of independent variables associated with RS

Characteristics	Intermediate vs low risk			High vs low risk		
	OR	95%CI	P value	OR	95%CI	P value
All patients(N=980)						
Grade						
I	1			1		
II	1.576	1.006~2.470	0.047	2.709	1.309~5.606	0.007
III	2.607	1.363~4.988	0.004	10.035	4.222~23.854	<0.001
T stage			NS			NS
PR			<0.001			<0.001
Positive	1			1		
Negative	4.397	1.952~9.901		12.039	5.207~27.836	
Ki67			NS			0.031
<14				1		
≥14				1.719	1.051~2.812	
Node-negative patients(N=874)						
Grade						
I	1			1		
II	1.662	1.041~2.655	0.033	2.724	1.303~5.694	0.008
III	2.463	1.241~4.889	0.010	9.730	3.994~23.701	<0.001
PR			<0.001			<0.001
Positive	1			1		
Negative	4.451	1.969~10.063		10.760	4.604~25.149	
Ki67			NS			NS
Node-positive patients(N=106)						
Grade			NS			NS
PR			NS			NS
Ki67			NS			NS

Abbreviations: PR, progesterone receptor; OR, odds ratio; CI, confidence interval; NS, not significant.

As for node-negative patients, both grade and PR status remained to be independent factors associated with RS. Conversely, no factors was identified to be correlated with RS in node-positive patients (Table 4).

### Relationship between IHC and RT-PCR results

**Figure 3:**
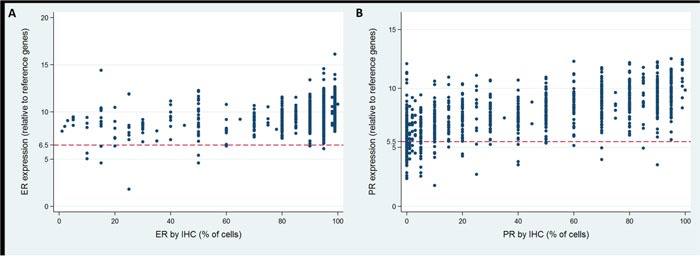
Concordance of estrogen receptor (ER) and progesterone receptor (PR) status between RT-PCR and IHC **(A)** ER; **(B)** PR.

IHC and RT-PCR of ER were concordant in 98.8% of all cases (98.7% and 99.1% in node-negative and node-positive patients, Table 5, Figure 3A). The correlation between IHC and RT-PCR of ER as a continuous variable was weak but statistically significant (all cases, r=0.362; node-negative cases, r=0.356; node-positive cases, r=0.418; all p<0.001). The Spearman's rank correlation between RS and ER IHC was -0.224, -0.213 and -0.328 in overall, node-negative and node-positive patients (all p<0.001).

**Table 5:** Relationship between IHC and RT-PCR results

	All patients			Node-negative patients			Node-positive patients		
	ER	PR	Ki67	ER	PR	Ki67	ER	PR	Ki67
Concordance between IHC and RT-PCR (%)	98.8	88.3	/	98.7	87.5	/	99.1	94.3	/
Spearman's correlation between IHC and RT-PCR	0.362	0.676	0.446	0.356	0.683	0.449	0.418	0.624	0.462
Spearman's correlation between IHC and RS	−0.224	−0.434	0.303	−0.213	−0.439	0.290	−0.328	−0.397	0.402

Abbreviations: IHC, immunohistochemistry; RT-PCR, reverse transcriptase-polymerase chain reaction; RS, recurrence score; ER, estrogen receptor; PR, progesterone receptor.

PR status between IHC and RT-PCR was concordant in 88.3% of all cases (87.5% and 94.3% in node-negative and node-positive patients, Table 5, Figure 3B). Comparing the continuous IHC and RT-PCR results of PR, a moderate relationship was observed (all cases, r=0.676; node-negative cases, r=0.683; node-positive cases, r=0.624; all p<0.001). Using Spearman's rank correlation, the relationship between RS and PR IHC results was calculated to be -0.434, -0.439 and -0.397 in overall, node-negative and node-positive patients (all p<0.001).

Regarding Ki67 index, there was a weak to moderate correlation between IHC and RT-PCR (all cases, r=0.446; node-negative cases, r=0.449; node-positive cases, r=0.462; all p<0.001, Table 5). The Spearman's rank correlation between RS and Ki67 IHC was 0.303, 0.290 and 0.402 in overall, node-negative and node-positive patients respectively (all p<0.001).

## DISCUSSION

The current study assessed the patterns of distribution of 21-gene RS in Chinese ER-positive, HER2-negative, node-negative or 1~3 node-positive breast cancer patients. To the best our knowledge, this study had a sample size larger than any others reported before regarding RS in Chinese population. In our study, RS was demonstrated to be significantly associated with grade, tumor size, PR status, Ki67 index and molecular subtype. In grade 3, T 2~3, PR-negative, high Ki67 and luminal-B tumors, the proportion to have a high-risk RS ranged from 31.0% to 48.3%, significantly higher than their counterparts. Similar to our findings, retrospective study of NSABP B-20 trial revealed that 42%, 30% and 51% patients with grade 3, T 2~3, PR-negative tumors were categorized as high-risk [4]. In PlanB trial, RS (low risk: RS≤11, intermediate risk: RS 12~25, high risk: RS≥26) was also found to be significantly associated with grade, PR status and Ki67 index [12]. Besides, the distribution of RS differed little across subgroups with different lymph node status. Multivariate analysis in our study indicated that poorly-differentiated, PR-negative or high-proliferating tumors had much higher probabilities to have increased RSs (grade 2/3 and negative-PR predictive for high and intermediate-risk RS, Ki67≥14% predictive for high-risk RS, Table 4). These facts indicated that routine clinical and pathologic parameters might be of help in predicting RS.

Discordance between clinic-pathologic factors and RS was also noted in our study. Some patients with favorable clinic-pathologic factors were found to display high-risk RS (e.g. 9.6% of patients with grade 1 tumors were high-risk). On the other hand, there were cases of tumors with low-risk RS that had unfavorable clinic-pathologic factors (e.g. 13.1% of patients with grade 3 tumors were low-risk). These results suggested that RS might provide not only integration of cancer-related genes and quantification of risk assessment, but also additional prognostic information beyond routine biologic parameters of individual breast cancers in Chinese patients.

Of note, the proportions of patients categorized as having a low, intermediate, or high risk were 26.1%, 49.3% and 24.6% in our study, different from that of the NSABP B-14 trial (low risk: 51%, intermediate risk: 22%, high risk: 27%). Similar findings were also reported in the latest studies of RS. In TAILORx trial, the first prospective validation study of RS, the proportion of patients having a low-risk RS was lower than previously reported results of the NSABP B-14 and B-20 trials, despite the different definition of risk categories [3, 4, 11]. Moreover, the distribution of scores observed by the commercial laboratory (Genomic Health) also differed from the initial report [11]. We noticed that the disparities of distribution mainly existed in low-risk and intermediate groups, whereas the proportion of high-risk RS in our study was similar to previous retrospective reports. As mentioned above, the proportions of high-risk RS in subgroups of unfavorable biologic markers (grade 3, T2~3, PR negative) in our study were quite comparable to that of the NSABP B-20 trial. We postulate that those discordance may be due to clinicians selecting patients for the enrolling into those clinical trials in whom there was therapeutic equipoise regarding the treatment benefit of experimental arms (e.g. extended tamoxifen group in NSABP B-14 trial and chemotherapy group in TAILORx trial). Conversely, we consecutively recruited eligible patients in our center in the past few years, which may be more close to actual distribution of risk groups in clinical practice. Furthermore, the distribution of RS might differ between Eastern and Western populations, and this inconsistent RS distribution needs further validation.

There has been growing interest in molecular biomarker testing by RT-PCR with conflicting results [15–20]. In our study, ER displayed a very high concordance of 98.8% between IHC and RT-PCR, in accordance with the literature (93% to 100%). Spearman's rank correlation showed that ER status by IHC had a positively weak relationship with ER by RT-PCR (r=0.362) and a negatively weak relationship with RS (r=-0.224). Previous studies reported that concordant cases had higher ER expression while discordant cases exhibited lower level of ER expression, which might partly account for the false-negative results by RT-PCR [15]. According to the present study, rarely ER by RT-PCR incorrectly determine ER status. Nonetheless, it's so far contraindicated to deny hormone therapies according to RT-PCR assay that would otherwise be indicated.

In the case of PR, a slightly lower concordance rate (88.3%) was detected than for ER. Similar concordance rates (87.7% to 94.2%) were reported in previous studies [15–20]. Meanwhile, spearman's rank correlation revealed that PR by IHC had a positively moderate relationship with PR by RT-PCR(r=0.676) and a negatively moderate relationship with RS(r=-0.434), higher than that of ER. This is comparable with some reports [15, 17–19], and lower than some others [16, 20]. Possible explanations for the lower concordance of PR status than ER was the change in the clones used to generate the ER and PR antibodies used for IHC and the positive staining criteria within the period of our study at our institution.

Limitations of this study could be the retrospective design and lack of follow-up. The follow-up visits is ongoing and it's immature to draw any conclusions regarding the prognostic significance of RS at this moment. Nonetheless, our study delineated the distribution patterns of RS across different biologic subgroups in Chinese ER-positive, HER2-negative early breast cancer patients for the first time, and explored the relationship of between molecular biomarker by IHC and 21-gene RT-PCR assay. Our findings appear to be plausible and warrant confirmation in further studies with long-term follow-up.

In Conclusion, 21-gene RS correlated significantly with tumor grade, T stage, PR status, Ki67 index and subtypes in Chinese ER-positive, HER2-negative early breast cancer patients. Grade, PR status and Ki67 index could independently predict RS. ER, PR status and Ki67 index between RT-PCR and IHC test had remarkable concordance.

## PATIENTS AND METHODS

### Study population

Nine hundred and eighty consecutive invasive breast cancer patients who underwent radical surgery between January 2009 and May 2016 in Shanghai Ruijin Hospital were retrospectively reviewed. Women with axillary node-negative or 1~3 nodes-positive breast cancer were eligible for inclusion if they had ER positive and HER2 negative tumors. Exclusion criteria were: T1a or T4 tumor, metastatic breast cancer, and previous neoadjuvant treatment. Following data were also collected: age at initial diagnosis, surgery type, pathology, histologic grade, progesterone receptor (PR) status and Ki67 index. Patients were subdivided into luminal A-like and luminal B-like subtypes according to 2013 St. Gallen Expert Consensus [21]. This retrospective study has been approved by the Ethical Committees of Shanghai Ruijin Hospital. The results of this study do not affect the treatment decision of any patient enrolled. All the clinical and pathological data was collected only after the written informed consent form was obtained from the patient.

### Analytic methods of recurrence score

The RS was determined from formalin-fixed, paraffin-embedded tissue as previously described [3]. In brief, hematoxylin and eosin-stained slides were reviewed to ensure sufficient invasive breast cancer (pathologist XC Fei), and then RNA was extracted from two 10μm unstained sections. Total RNA content was measured, and the absence of DNA contamination was verified. Gene-specific reverse transcription was performed followed by standardized quantitative RT-PCR reactions in 96 well plates using Applied Biosystems (Foster City, CA) 7500 Real-Time PCR System. Expression of each gene was measured in triplicate, and normalized relative to a set of five reference genes. Reference-normalized expression measurements range from 0 to 15, and a 1-unit increase reflected approximately a two-fold increase in RNA. A tumor is considered ER-negative if expression units <6.5, ER-positive ≥6.5, PR-negative <5.5, PR-positive ≥5.5 [15]. The RS, ranging from 0 to 100, was derived from the reference-normalized expression measurements for the 16 cancer-related genes. Patients were then categorized into low-risk (RS<18), intermediate-risk (RS 18~30), and high-risk (RS≥31) groups [3].

### Immunohistochemistry (IHC)

Positive staining for ER/PR was defined as nuclear staining in ≥1% of the tumor cells. Negative HER2 status was considered as 0 to 1+ by IHC or negative on FISH. Ki-67 index was characterized as the proportion of positively nuclear staining cells among at least 1000 tumor cells in the area counted. The following antibodies were used for the IHC test: ER: clone 1D5 (rabbit monoclonal, Gene), PR: clone PR636 (mouse monoclonal, Dako), HER2: 4B5 (rabbit monoclonal, Roche), Ki67: MIB-1 (mouse monoclonal, Dako).

### Statistical analysis

The Chi-square test were applied to evaluate the distribution of RS risk category among patients with different clinico-pathologic factors. Fisher's exact test was performed when necessary. Multivariate logistic regression analysis was used to estimate the odds ratio (OR), 95% confidence interval (CI), and the association between clinical and pathological variables and RS. Spearman's rank correlation was used to compare the results of ER, PR and Ki67 on RT-PCR and IHC. All statistical tests were two sided and p<0.05 was considered significant. The software package STATA (version 14.0, College Station, TX, US) for Windows 10 was used for analysis.
